# Response to: Trigeminal nerve chronic motor denervation caused by cerebellar peduncle pilocytic astrocytoma

**DOI:** 10.1007/s00381-023-06230-6

**Published:** 2023-12-22

**Authors:** Giada Del Baldo, Valerio Cecinati, Giovanna Stefania Colafati, Andrea Carai, Angela Mastronuzzi

**Affiliations:** 1 Department of Pediatric Haematology and Oncology, Cell and Gene Therapy, Bambino Gesù Children’s Hospital, IRCCS, Rome, Italy; 2 Department of Experimental Medicine, Sapienza University of Rome, Rome, Italy; 3 Complex Structure of Pediatrics and Pediatric Oncohematology, “Nadia Toffa”, Central Hospital Santissima Annunziata, Taranto, Italy; 4 Department of Diagnostic Imaging Oncological Neuroradiology Unit, Bambino Gesù Children’s Hospital, IRCCS, Rome, Italy; 5 Neurosurgery Unit, Department of Neurosciences, Bambino Gesù Children’s Hospital, IRCCS, Rome, Italy

Dear Editor,

With great interest, we have read the work published by Papangelopoulou et al. [1] in which they report the rare clinical finding of trigeminal chronic motor denervation caused by cerebellar peduncle pilocytic astrocytoma in a child.

**Fig. 1:**
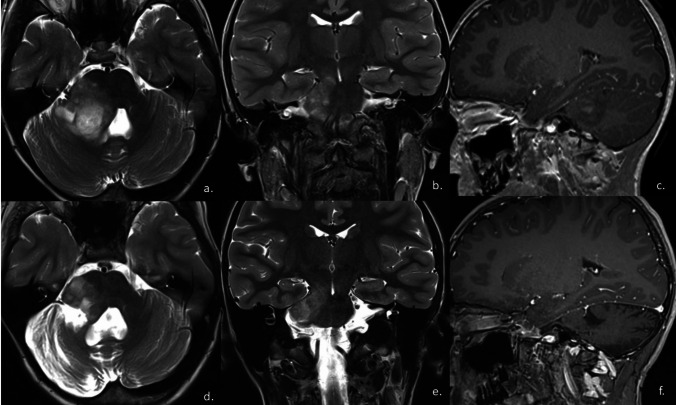
Axial (**a**, **d**) and coronal (**b**, **e**) T2w images and sagittal Gd T1w images (**c**, **f**). At onset (upper row), there is solid formation with cystic components localised in the right ponto-cerebellar region with involvement of the root entry zone (REZ) of the trigeminal nerve. In sagittal T1 images, inhomogeneous contrast-enhancement of the lesion and slightly thin appearance of the right trigeminal nerve in the cisternal tract in relation to the contralateral are evident. Follow-up (bottom row) at 60 months shows stable disease with no significant change in residual disease in the REZ region nor in the appearance of the trigeminal nerve

We describe a similar case of a female patient who came to our attention at the age of six, with a 3-year history of progressive weakness of the right lower limb, mainly during physical activity. The condition clinically progressed with the onset of paroxysmal pain and paresthesia on the right side of the face. Magnetic resonance imaging performed upon arrival at our center documented the presence of a tumor in the right middle cerebellar peduncle, extending ventrally into the right half of the pons and midbrain, reaching the homolateral cerebral peduncle. There was also a dorsal lateral extension into the right cerebellar cortical area. The lesion appeared heterogeneously hyperintense in long TR images and hypointense in T1, with the presence of some areas with a cerebrospinal fluid-like signal, possibly due to partially cystic components, the largest of which was in the cranial and dorsal portion (Fig. 1).

Detailed physical examination documented motor impairment, hypoesthesia on the right side of the face and pain at palpation of emergence points of the homolateral trigeminal nerve branches. The paroxysmal pain topographical distribution could be attributed to involvement of the first and second branches of the right trigeminal nerve.

The patient underwent occipital craniotomy and subtotal resection of the neoplasm, which turned out to be a pilocytic astrocytoma, and underwent follow-up evaluations that consistently showed stability of the residual tumor tissue until now.

Following the surgical removal, the child showed an improvement in lower limb motor impairment, which almost completely recovered after intensive rehabilitation and the development of compensatory strategies. However, despite initial improvement of trigeminal symptoms and evidence of stable residual disease, there was a progressive worsening of trigeminal neuralgia, necessitating chronic pharmacological therapy with carbamazepine. This painful symptomatology was associated to progressive atrophy of the homolateral masticatory muscles, leading to facial asymmetry.

We believe that involvement of motor nucleus of the trigeminal nerve, although rare, should always be investigated in patients with intraaxial tumors arising from the middle cerebellar peduncle and extending ventrally [2]. Since the motor nucleus is located ventro-medially to the sensitive nuclei, the possibility of associated sensibility impairment, including trigeminal neuralgia, should always be suspected, especially in infants unable to correctly report sensibility symptoms. Pilocytic astrocytoma is a grade I brain tumor according to 2021 WHO classification [3]. Surgical removal remains the cornerstone of treatment; other therapies are indicated in cases of disease-related clinical complication and/or significant tumor progression on MRI [4]. Despite the possibility of a good prognosis of the neoplasm, when there is involvement of trigeminal nerve, this could have a detrimental impact on the patient’s quality of life in terms of pain and the appearance of facial dysmorphisms [5].
